# Primary vulval melanoma and genital lichen sclerosus

**DOI:** 10.1002/ski2.411

**Published:** 2024-06-25

**Authors:** Evanthia Mastoraki, Georgios Kravvas, Kate Dear, Sharmaine Sim, Mariel James, Richard Watchorn, Aiman Haider, Peter Ellery, Alex Freeman, Mahfooz Basha, Emma Edmonds, Christopher B. Bunker

**Affiliations:** ^1^ Department of Dermatology University College London Hospitals NHS Foundation Trust London UK; ^2^ Department of Medicine University College London Medical School London UK; ^3^ Department of Histopathology University College London Hospitals NHS Foundation Trust London UK

## Abstract

**Background:**

Lichen sclerosus (LS) is a chronic, inflammatory skin disease with a predilection for the genitalia. Although, the association between squamous cell cancer and genital LS is well established, a link with genital melanoma has not been thoroughly explored. However, we have recently published a case series of penile melanoma where 9/11 (82%) of patients seen over a 10 year period with penile melanoma were retrospectively found to have histological and/or clinical evidence of genital LS on review.

**Objectives:**

The aim of this study was to illuminate further the relationship between vulval melanoma and genital LS by reviewing all the cases managed by our hospital and undertaking a literature review.

**Methods:**

We identified all the cases with a diagnosis of vulval melanoma over a 16‐year period (2006–2022) where histology was available. The clinical notes were retrospectively reviewed, and the histological features of all cases were reassessed by two independent mutually ‘blinded’ histopathologists. We also performed a literature review of genital LS in patients with vulval melanoma.

**Results:**

A total of 11 patients with vulval melanoma were identified for the review. Histopathological review found evidence of genital LS in seven of them (64%). Genital LS was not documented in any of the original histology reports. Clinical notes and letters were available in nine cases. The literature review identified 12 relevant studies with a total of 18 patients. Twelve cases concerned adult women, and six concerned female children.

**Conclusion:**

The presence of genital LS in as high as 64% of our vulval melanoma cases might indicate a causative relationship between genital LS and vulval melanoma. The pathogenesis of vulval melanoma remains largely unknown. Although ultraviolet radiation is an important pathogenic factor for cutaneous melanoma, it cannot be a factor in vulval melanoma. While possible mechanisms behind this association remain unclear, it is possible that chronic inflammation from genital LS leads to melanocytic distress and increased mutagenesis.

**What is already known?**

- Vulval melanoma is an uncommon condition with a poor prognosis that accounts for up to 10% of all primary vulval malignancies and for 1%–2% of all melanomas in females.
- Although, the association between squamous cell cancer (SCC) and genital lichen sclerosus (LS) is well established, a link between LS and genital melanoma has not been thoroughly explored.

**What does this study add?**

- This study highlights that the presence of genital LS in as high as 64% of our vulval melanoma cases might indicate a causative relationship between genital LS and vulval melanoma.
- While the mechanisms behind the association of genital LS and vulval melanoma remain unclear, our findings have implications for the early diagnosis, management and follow‐up of genital LS.

## 1 INTRODUCTION

Lichen sclerosus (LS) is a chronic inflammatory skin disease with a predilection for the genitalia. Although, the association between squamous cell cancer (SCC) and genital LS is well established, a link with genital melanoma has not been thoroughly explored.^1^, ^2^, ^3^, ^4^

Recently, we reviewed the literature pertaining to male genital LS and penile melanoma and published a case series where 9/11 (82%) of patients with penile melanoma were found to have histological and/or clinical evidence of genital LS.^5^, ^6^

However, recent reports in the paediatric and vulval literature also suggest a possible association between genital LS and vulval melanoma.^7^, ^8^, ^9^, ^10^, ^11^, ^12^, ^13^, ^14^, ^15^, ^16^, ^17^, ^18^

## 2 METHODS

The aim of this study was to explore further the relationship between vulval melanoma and genital LS.

We retrospectively identified all cases of vulval melanoma managed in our department over a 16‐year period for which histopathological material was available (2006–2022). The histological features of all cases were reviewed by two independent, mutually ‘blinded’ histopathologists. In cases of diagnostic discordance between the two histopathologists, the slides were further reviewed by a third independent histopathologist.

Additionally, we performed a literature search on articles and included those that mentioned cases with both vulval melanoma and vulval LS.

## 3 RESULTS

A total of 11 patients were identified. The median age at diagnosis of vulval melanoma was 64 years (range 48–87 years) and the majority of patients (73%) were White British. The most common subtype of melanoma seen was superficial spreading (seven cases, 64%), with nodular melanoma recorded in three cases (27%,) and mucosal melanoma in one case (9%). Cancer staging was found to be as follows: pTis in three cases (30%), pT2a in two cases (20%), pT2b (10%) in one case, pT3a in one case (10%), pT4a in one case (10%) and PT4b in two cases (20%). Staging was not available in one case. KIT mutation was only identified in one case.

Significantly, LS was not mentioned in the clinical records of any patient, no clinical photographs were available for review, and LS was not mentioned in any of the original histology reports.

**FIGURE 1:**
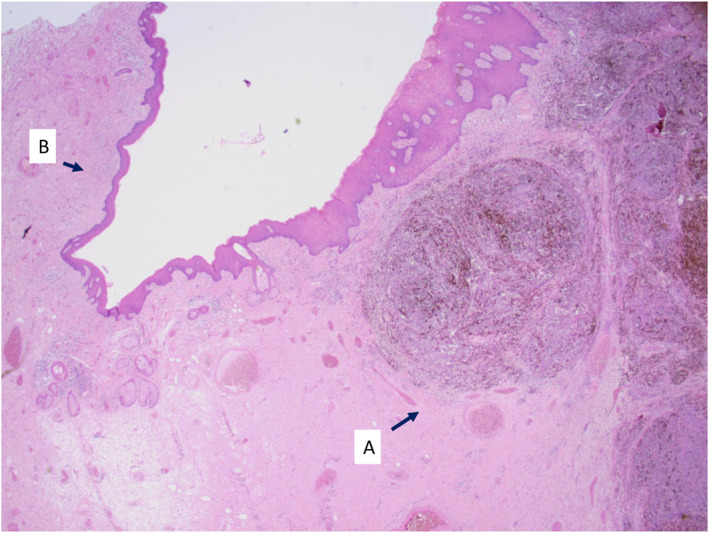
Vulval excision for melanoma (haematoxylin and eosin, original magnification x2). (a) Infiltrating nodules of invasive melanoma extending into the dermis; (b) the adjacent surface epithelium shows epidermal atrophy, loss of skin adnexal structures, fibrosis in the superficial dermis and a lichenoid band of chronic inflammation in keeping with lichen sclerosus.

**FIGURE 2:**
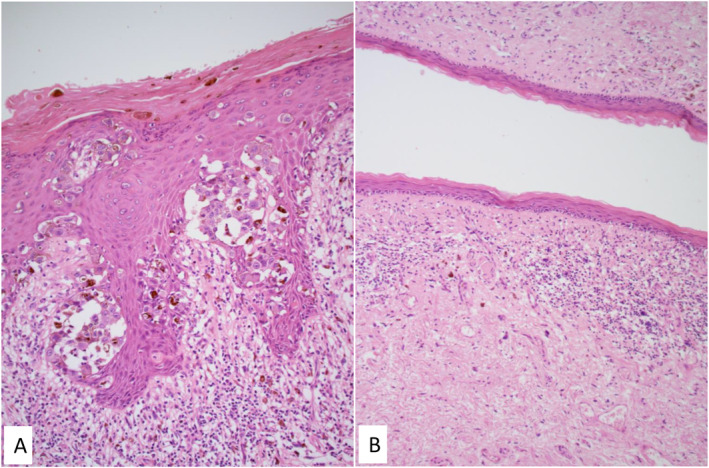
Vulval excision for melanoma. (a) Superficially spreading melanoma containing multiple clusters of atypical melanocytes in the epidermis (haematoxylin and eosin, original magnification x40); (b) a section from the background skin from the same patient showing features in keeping with lichen sclerosus (epidermal atrophy, loss of rete ridges, lichenoid band of inflammation and subepithelial sclerosis) (haematoxylin and eosin, original magnification x10).

Overall, genital LS was histologically present in seven cases (64%) and absent in four (36%) (see Figures 1 and 2). Histopathological findings for genital LS were concordantly positive in one case and concordantly negative in four cases. In the six cases in which histopathological opinions were discordant, a third histopathologist reviewed the cases and deemed genital LS to be present in all six.

The literature review of the topic identified 12 relevant studies with a total of 18 patients. Twelve cases concerned adult women, and six concerned female children.^7^, ^8^, ^9^, ^10^, ^11^, ^12^, ^13^, ^14^, ^15^, ^16^, ^17^, ^18^

## 4 DISCUSSION

Vulval melanoma is an uncommon condition with a poor prognosis that accounts for up to 10% of all primary vulval malignancies and for 1%–2% of all melanomas in females.^1^, ^4^, ^19^, ^20^, ^21^, ^22^, ^23^ Although rare, vulval melanoma is the second commonest form of vulval cancer after SCC of the vulva, which represents up to 90% of all vulval malignancies.^1^, ^19^, ^20^, ^21^

The majority of vulval melanomas occur in post‐menopausal Caucasian women with a median age at diagnosis between 56 and 68 years, but have also been reported in paediatric patients.^7^, ^8^, ^9^, ^10^, ^11^, ^12^, ^23^, ^24^, ^25^, ^26^, ^27^, ^28^, ^29^ Similarly, penile melanoma is a rare entity that accounts for less than 1.4% of all primary penile cancers, and less than 0.1% of all melanomas in the male sex and mainly affects older men.^30^

Several pathogenic factors have been studied for their role in vulval melanoma, including viruses [*e.g*. human papilloma viruses, human herpes viruses and polyomavirus], chronic inflammatory diseases and chemical irritants, but no conclusive correlations have yet been drawn.^4^, ^12^, ^18^, ^22^, ^31^, ^32^, ^33^, ^34^, ^35^

Vulval melanoma usually affects the glabrous (non‐hair‐bearing) parts of the vulva, most commonly the labia minora, the labia majora and clitoris.^4^, ^16^, ^19^, ^24^, ^36^, ^37^

Vulval LS is a chronic progressive inflammatory skin disorder that primarily involves the glabrous skin.^1^, ^38^ Although a number of aetiological mechanisms have been proposed (including genetic, hormonal, irritant, traumatic and infectious factors), the exact aetiopathogenesis remains controversial.^38^

It is now well recognized that women with vulval LS have an increased risk for developing vulval SCC.^1^, ^38^, ^39^ The estimated lifetime risk of vulval SCC in patients with genital LS ranges from 2.2% to 5%.^1^, ^39^, ^40^ However, this has not been quantified in untreated patients or in relation to the length of time that the disease has been present.^41^ It is believed that early detection and treatment can lead to reduction in the risk of developing SCC.^39^, ^41^

The literature contains an increasing number of case reports of vulval melanomas associated with genital LS, but a definitive relationship between them has not yet been established.^4^, ^5^, ^6^, ^7^, ^8^, ^9^, ^10^, ^11^, ^12^, ^13^, ^14^, ^15^, ^16^, ^17^, ^18^ To our knowledge, there have been 18 previously published cases of vulval melanomas coexisting with genital LS and 12 cases of penile melanomas linked to genital LS.^5^, ^6^, ^7^, ^8^, ^9^, ^10^, ^11^, ^12^, ^13^, ^14^, ^15^, ^16^, ^17^, ^18^ Also, it should be noted that there are no case reports of vulval melanomas in juveniles without LS.^42^, ^43^

A study in 2018 based on data extracted from the Finnish Cancer Registry found 249 patients with biopsy‐confirmed vulval LS, of whom three also had vulval melanomas.^14^ From the same registry, 30 cases of SCC were found among patients with vulval LS. Conversely, six cases of melanomas were identified among patients who did not have genital LS from a total of 250 000 females. The overall risk of vulval melanoma for patients with vulval LS was thus estimated to be 341 times higher than the risk for patients without vulval LS (*p* < 0.001).^14^

Our findings are in line with these publications. Recent work by our group reported evidence of LS in 82% of all penile melanomas.^5^ The rates of genital LS found in this study in females are lower (64%), but they still support an association. Comparing the prevalence of vulval LS in the general population (0.1%–1.7%) with the prevalence of vulval LS in vulval melanoma demonstrated in this review (64%) gives perspective to the strength of the correlation.^44^

We thus argue that the actual prevalence of genital LS in vulval melanoma is higher than that which is reflected in the existing literature.

It seems likely that the striking histological features of vulval melanoma overshadow the more subtle features of genital LS, so that genital LS is under‐recognized and under‐reported, analogous to the situation observed in genital skin adjacent to SCC, and thus only detected when determinedly sought out, as in this case series.^5^, ^12^, ^45^, ^46^

In addition, the observation that the histological features of LS appear to disappear under melanoma, but not under naevi, might contribute to the difficulty in the diagnosis of LS in this context.^5^, ^12^

Genital skin is largely unexposed through life to UV radiation—the predominant environmental melanocytic carcinogen. UV cannot therefore be credibly considered a causative factor in vulval melanoma. It seems more plausible, instead, that in genital skin local stressors and defective mechanisms of local tissue homoeostasis are responsible for the development of melanoma.^47^

It is possible that molecular events involved in squamous carcinogenesis in genital LS to SCC are also relevant in the development of melanoma. But they might equally be independent.

Some of the pathological mechanisms implicated in genital LS involve immunogenic activation, sclerosis and oxidative stress.^38^, ^39^

Genital LS may be responsible for generating a pro‐oxidative environment that increases the risk of mutations whilst also altering the extra‐cellular matrix composition, leading to clonal expansion of damaged melanocytes.^5^, ^12^ Keratinocytes and melanocytes overlying scars have been found to exhibit an ‘activated’ immunophenotype denoted by increased keratinocytic proliferation, increased HMB‐45 expression and increased proliferation of melanocytes.^12^, ^48^, ^49^, ^50^ LS could be exhibiting a regenerative phenotype as a response to stromal fibrosis and sclerosis, whist also preventing the function of T‐cytotoxic T lymphocytes allowing for the expansion of melanocytes with an abnormal phenotype.^12^

Oxidative stress contributes to inactivation of tumour suppressor genes (such as p53 and CDKN2A), leading to cell proliferation and malignant transformation.^51^, ^52^, ^53^

Soufir et al. demonstrated that inactivating mutations of the *p53* and *CDKN2A* genes were present in 100% of LS‐derived genital tumours. Although, they seemed to occur later in oncogenesis as they were not present in the initial LS lesions. In addition, an overexpression of the p53 wild‐type protein has been observed in LS, reflecting stress response to inflammation and carcinogenic insults.^52^

Several studies suggest that *C‐KIT, TP53* and *NRAS* mutations are seen with high frequency in vulval melanoma. BRAF mutations in vulval melanomas seems to be less frequent, compared to cutaneous melanomas, probably due to the absence of UV mutational signature in vulval melanomas.^22^, ^54^, ^55^

Melanocytic dysfunction and distress are commonly seen during and after genital LS, manifesting as benign vulval melanosis, and there is a literature attesting to the challenges this can create in the differential diagnosis of these lesions.^2^, ^12^, ^49^, ^56^ For this reason, we need to be careful when considering vulval melanoma in a background of LS, especially in children and adolescents.^42^, ^49^ Melanocytic hyperplasia has been documented in LS, but it is generally rare and difficult to interpret. Benign melanocytic naevi can be encountered in genital LS that sometimes histologically resemble melanoma.^6^, ^12^, ^49^, ^56^, ^57^ It is not clear whether melanocytic lesions (such as naevi or vulval melanosis) were present in any of our cases prior to the development of the malignant lesion. However, it is possible that LS could also induce the formation of a melanocytic nevus from either/or predisposed melanocytes, an activated mesenchyme and pathologically changed extracellular matrix of LS.^12^

This study is limited by its size and the retrospective collection of data, and the lack of clinical photographs. It can be argued that the histopathology findings may have been distorted by subjectivity. We attempted to mitigate this possibility by engaging two histopathologists to review the histology independently, with a third histopathologist recruited to address any discordant findings.

## 5 CONCLUSION

The histological presence of genital LS in seven out of 11 patients with vulval melanoma raises the hypothesis of a causative relationship between the two conditions. We postulate that the limited number of reports on this topic are related to clinical and histological under‐recognition and under‐reporting of genital LS. It is suggested that chronic inflammation from genital LS leads to melanocytic distress and increased mutagenesis. This notion is further supported by the absence of UV mutational signatures in vulval melanomas and the anatomical and multi‐focal presentation of vulval melanomas, which mirrors that of genital LS. While the mechanisms behind this association remain unclear, our findings have implications for the early diagnosis, management and follow‐up of genital LS.

## CONFLICT OF INTEREST STATEMENT

None to declare.

## AUTHOR CONTRIBUTIONS

**Evanthia Mastoraki**: Conceptualization (equal); data curation (equal); formal analysis (equal); investigation (equal); methodology (equal); validation (equal); visualization (equal); writing – original draft (equal); writing – review & editing (equal). **Georgios Kravvas**: Conceptualization (equal); data curation (equal); formal analysis (equal); investigation (equal); methodology (equal); project administration (equal); supervision (equal); validation (equal); visualization (equal); writing – original draft (equal); writing – review & editing (equal). **Kate Dear**: Data curation (equal); formal analysis (equal); investigation (equal); methodology (equal); validation (equal). **Sharmaine Sim**: Data curation (equal); formal analysis (equal); investigation (equal); methodology (equal); validation (equal). **Mariel James**: Data curation (equal); formal analysis (equal); investigation (equal); methodology (equal); validation (equal). **Richard Watchorn**: Data curation (equal); formal analysis (equal); investigation (equal); methodology (equal); validation (equal). **Aiman Haider**: Conceptualization (equal); data curation (equal); formal analysis (equal); investigation (equal); methodology (equal); validation (equal); visualization (equal); writing – original draft (equal); writing – review & editing (equal). **Peter Ellery**: Conceptualization (equal); data curation (equal); formal analysis (equal); investigation (equal); methodology (equal); supervision (equal); validation (equal); visualization (equal). **Alex Freeman**: Data curation (equal); formal analysis (equal); investigation (equal); methodology (equal); validation (equal). **Mahfooz Basha**: Data curation (equal); formal analysis (equal); investigation (equal); methodology (equal); validation (equal). **Emma Edmonds**: Data curation (equal); formal analysis (equal); investigation (equal); methodology (equal); validation (equal). **Christopher B. Bunker**: Conceptualization (equal); data curation (equal); formal analysis (equal); investigation (equal); methodology (equal); supervision (equal); validation (equal); visualization (equal); writing – original draft (equal); writing – review & editing (equal).

## ETHICS STATEMENT

Not applicable.

## PATIENT CONSENT

Not applicable.

## Data Availability

The data underlying this article will be shared on reasonable request to the corresponding author.
